# Outbreak of colonizations by extended-spectrum β-lactamase-producing *Escherichia coli* sequence type 131 in a neonatal intensive care unit, Italy

**DOI:** 10.1186/2047-2994-2-8

**Published:** 2013-03-21

**Authors:** Mario Giuffrè, Domenico Cipolla, Celestino Bonura, Daniela Maria Geraci, Aurora Aleo, Stefania Di Noto, Federica Nociforo, Giovanni Corsello, Caterina Mammina

**Affiliations:** 1 Department of Sciences for Health Promotion and Mother-Child Care “G. D’Alessandro”, University of Palermo, Via del Vespro 133, I-90127, Palermo, Italy; 2 PhD School in Food and Human Nutrition, University of Palermo, Palermo, Italy; 3 Specialty School in Pediatrics, University of Palermo, Palermo, Italy

**Keywords:** ESBL-*Escherichia coli*, ST131, NICU, Epidemiology

## Abstract

**Background:**

Extended spectrum β-lactamases (ESBLs) often associated with resistance to aminoglycosides and fluoroquinolones have recently emerged in community-associated *Escherichia coli*. The worldwide clonal dissemination of *E. coli* sequence type (ST)131 is playing a prominent role.

We describe an outbreak of colonizations by ESBL-producing *E. coli* (ESBL-*E. coli*) in the neonatal intensive care unit (NICU) of the University Hospital, Palermo, Italy.

**Methods:**

An epidemiological investigation was conducted with the support of molecular typing. All children admitted to the NICU and colonized by ESBL-*E. coli* between January and June 2012, were included in the study. Cases were defined as infants colonized by *E. coli* resistant to third generation cephalosporins and fluoroquinolones. A case–control study was also performed to identify possible risk factors.

**Results:**

During the outbreak period, 15 infants were found to be colonized by ESBL-*E. coli*. The epidemic strain demonstrated continuous transmission throughout the outbreak period. Case–control study identified a lower birth weight as the only risk factor for colonization. The strain belonged to the sequence-type 131 community-associated clone. Transmission control interventions, including contact precautions and cohorting, restriction of the new admissions, sanitization of surfaces and equipment and targeted training sessions of the NICU staff, were successful in interrupting the outbreak.

**Conclusions:**

Although invasive infections did not develop in any of the 15 colonized neonates, our report highlights the need to strictly monitor the spill in the NICU setting of multidrug resistant community-associated organisms. Our findings confirm also the role of active surveillance in detecting the silent spread of ESBL-producing Gram negatives in a critical healthcare setting and trigging the implementation of infection control measures. As β-lactam and fluoroquinolone resistant *E. coli* strains are increasingly spreading in the community, this event could become a more serious challenge.

## Introduction

*Escherichia coli* is a common cause of community and healthcare associated infections. Over the past few decades, *E. coli* strains isolated from community-acquired infections have become increasingly resistant to antibacterial drugs [1]. Recently, extended spectrum β-lactamases (ESBLs) often associated with resistance to aminoglycosides and fluoroquinolones have emerged in community-associated *E. coli*[1,2]. The worldwide clonal dissemination of some successful strains, such as *E. coli* sequence type (ST)131, is playing a prominent role [1].

The identification of ESBL-producing *E. coli* (ESBL-*E.coli*) as causative agents of infections in neonatal wards is a worrying epidemiological development, because of the serious limitations to inherently restricted therapeutic options. On the other hand, these healthcare settings are proving to be particularly prone to the invasion by community associated multidrug resistant (MDR) organisms, due to their peculiar position at the interface between hospital and community [3-6].

We report here an outbreak of colonizations by an ESBL-*E.coli* strain belonging to ST131 in the neonatal intensive care unit (NICU) of the University Hospital in Palermo, Italy.

## Methods

### Epidemiological investigation

During the first semester of 2012, an unexpected increase in the frequency of isolation of ESBL-*E. coli* was observed in the NICU of the University Hospital “Azienda Ospedaliero-Universitaria Policlinico P. Giaccone”, Palermo, Italy. The NICU annually admits 250 infants approximately of all gestational ages (mean 36.7, range 26–42). Because it is associated to the regional reference centre for genetic diseases, the NICU has a high prevalence of neonates with malformations (20% approximately) as well as of outborn admissions (35% approximately). Moreover, a further 20% proportion of patients has complex conditions requiring subspecialty medical or surgical care. The NICU includes one intensive care room consisting of 8 beds and one intermediate care room including 8 further beds. The average nurse to patient ratio is 1:3 to 1:4 in the two sections, respectively.

Since June 2009, a surveillance protocol is routinely in place which includes rectal swabs obtained on a weekly basis (every Tuesday) from all infants staying in the NICU to monitor the prevalence of colonization with multidrug resistant (MDR) Gram negatives.

All children admitted to the NICU and colonized by ESBL-*E. coli* between January and June 2012, were included in the study. Cases were defined as infants colonized by *E. coli* simultaneously resistant to third generation cephalosporins and fluoroquinolones. Microbiology database was queried as far back as January 2010 to identify additional ESBL-*E. coli* isolates.

The study protocol was approved by the Ethics Committee of the Azienda Ospedaliero-Universitaria Policlinico “P. Giaccone”, Palermo, Italy, and informed consent was sought in accordance with the principles of the Declaration of Helsinki. Written informed consent was obtained from the parents of each patient.

### Case–control study

A case–control study was performed to look for possible risk factors for the colonization by the ESBL-*E.coli* strain. Controls were the infants who were being admitted between the admission of the index case and the discharge of the last case and had negative surveillance cultures for ESBL-*E.coli*. For cases, risk factors were assessed for the interval between admission and the first positive culture for ESBL-*E. coli*. For controls, risk factors were assessed for the entire NICU stay. Data on demographics, way of delivery, gestational age, birth weight, singleton or twin pregnancy, antibiotics administration, surgical procedures, exposure to devices and length of stay were collected. Univariate analysis was carried out by the EpiInfo software (ver.7; Centers for Disease Control and Prevention, Atlanta, GA, US). Tests of significance were performed with χ^2^ or Student’s *t* test where appropriate. The assessment of certain risk factors such as birth weight, length of stay, device exposures, and length of antibiotic treatment, analysis of variance (ANOVA) was used. Stepwise logistic regression analysis of variables found significant in the univariate analysis (P <0.10) was performed using StatPlus 2009 software. All significance tests were two-tailed, and *P* <0.05 was considered significant.

### Microbiological investigations

Intestinal colonization by antibiotic-resistant Gram negatives was assessed by culturing broth-enriched rectal swabs onto MacConkey agar. Four antibiotic disks, containing gentamicin (10 μg), amoxicillin-clavulanic acid (20–10 μg), meropenem (10 μg) and ceftazidime (30 μg) were placed on each plate before incubation. These antimicrobial drugs were selected in order to include the two more frequently used antibacterial drugs in UTIN, gentamicin and amoxicillin-clavulanic acid, and two further antibiotics, such as ceftazidime and meropenem, that could allow for timely detecting Gram negative organisms resistant to cephalosporins and/or carbapenems. Selective media were not employed, so that Gram negative colonization could be detected and ceftazidime-susceptible and –non susceptible organisms could be isolated. Colonies growing into antibiotic inhibition halos were subcultured, biochemically identified using API20E strips (Biomérieux, Marcy l'Étoile, France) and submitted to antibiotic susceptibility testing [7]. Antibiotic susceptibility testing and ESBL detection were first performed by disk diffusion and double disk synergy test and then confirmed by Etest (Biomérieux, Marcy l'Étoile, France) methods according with the European Committee on Antimicrobial Susceptibility Testing (EUCAST) guidelines [8]. An isolate was defined as resistant to third generation cephalosporins, when the inhibition zone diameter of ceftriaxone (30 μg) and cefotaxime (5 μg) was < 20 mm and 17 mm, respectively [8]. This isolate was defined as simultaneously resistant to fluoroquinolones when the inhibition zone diameter of ciprofloxacin (5 μg) was < 19 mm [8]. *E. coli* ATCC 25922 was used as quality control strain. An alert email was sent to NICU in the event of growth of colonies or no inhibition zone around the ceftazidime and/or meropenem disk.

*E*. *coli* phylogenetic group was determined by a triplex polymerase chain reaction (PCR) assay as described previously [9].

Multiplex PCRs were used to amplify genes encoding ESBLs [10]. *bla*_CTX-M_ genes were amplified with specific primers and sequenced. In addition, the *aac*(6’)-*Ib-cr* aminoglycoside-quinolone resistance gene was characterized by PCR amplification and sequencing. Conjugation experiments were performed using the *E. coli* strain K12J5 Rif^r^ as a recipient. Plasmids were classified according to their incompatibility group by using a PCR-based replicon typing scheme [11].

Clonality of the ESBL-*E. coli* isolates was determined by rep-PCR using the DiversiLab *Escherichia* kit for DNA fingerprinting (BioMerieux, Marcy-l'Etoile, France) [12]. *E. coli* isolates were further characterized by multilocus sequence typing (MLST) according to the University College Cork (Cork, Ireland) scheme for *E. coli* (http://mlst.ucc.ie/mlst/dbs/Ecoli).

## Results

**Figure 1:**
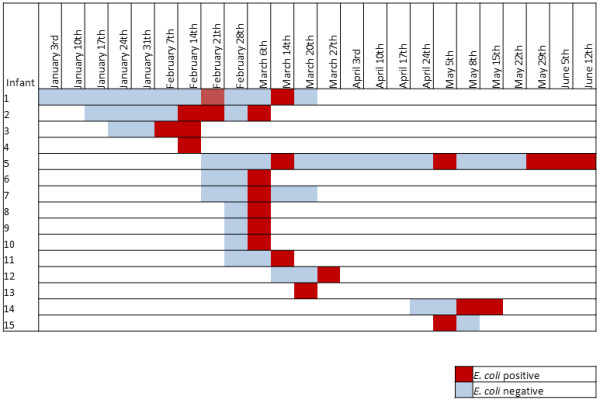
**ESBL-producing *Escherichia coli *colonizations during the outbreak period.**

Between February 7 and June 12, 2012, 15 infants were found to be colonized by ESBL-*E. coli* (Figure 1). In this interval of time, 103 newborns were admitted to the NICU. Reviewing of the microbiology database showed five ESBL-*E.coli* isolations in 2010 and two in 2011. However, no clustering was evident.

**Figure 2:**
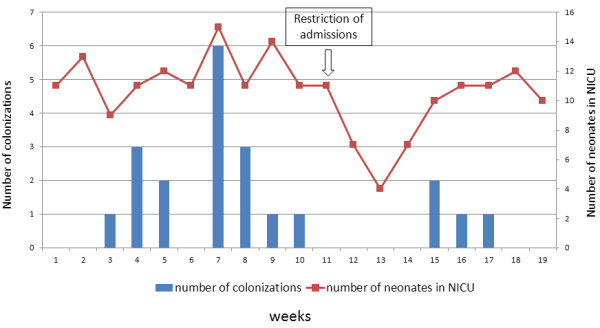
**Pattern of transmission of ESBL-*Escherichia coli *among the colonized infants in NICU during the outbreak period.**

The index case was a preterm infant with congenital hydrocephalus born at 35 weeks gestational age, who underwent surgical ventriculo-peritoneal derivation at three days of life and antibiotic therapy with ampicillin-sulbactam and gentamicin for the first 2 weeks of life. Detection of ESBL-*E. coli* occurred after 18 days since his admission to the NICU. Until March 27, 12 additional cases of colonization were detected, two of whom had been admitted before the index case (Table 1). The pattern of transmission of ESBL-*E. coli* is shown in Figure 2. The epidemic strain demonstrated continuous transmission throughout the outbreak period. Three infants were found to intermittently carry ESBL-*E.coli*. This was assumed as a persistent colonization because clearance followed by the reacquisition of the same strain was thought to be unlikely to have occurred.

**Table 1:** **Characteristics of the infants colonized by ESBL-*E. coli *and risk factors at admission and during the NICU stay**

**Infant**	**Date of admission**	**Gestational age, week / birth weight, g.**	**Type of delivery**	**Lengh of stay (days)**	**Days in unit prior to first positive rectal swab**	**Feeding type**	**Status at the discharge time**
1	December 27^b^	27/1140	CD	88	78	mixed	alive
2	January 16	30/1110	CD	54	29	mixed	alive
3^a^	January 20	35/2420	CD	31	18	formula	transferred to a surgical ward
4	February 10	36/1970	CD	8	4	formula	alive
5	February 16	30/720	CD	121	27	mixed	alive
6	February 18	33/1910	CD	33	17	mixed	alive
7	February 19	34/2270	VD	16	16	breast milk	alive
8	February 22	38/2680	CD	16	13	formula	alive
9	February 22	31/1790	VD	22	20	formula	alive
10	February 23	37/1850	CD	12	12	mixed	alive
11	February 27	38/u.i.^c^	VD	11	8	formula	alive
12	March 9	35/3300	CD	25	18	formula	alive
13	March 19	40/3440	CD	3	1	formula	alive
14	April 17	41/3000	CD	30	19	mixed	alive
15	April 28	35/1840	CD	14	10	mixed	alive

CD, cesarean delivery; VD, vaginal delivery; ^a^index case; ^b^ 2011; ^c^unavailable information.

Contact precautions were implemented when screening samples tested positive for ceftazidime resistant organisms. The decision was also made to restrict the admission of new patients. However, a third small cluster of colonization cases involving two new patients occurred one month after the last colonization case was detected. Only one colonized infant was staying in the NICU through this interval of time (no.5, Figure 2). This infant was also the last colonized patient to be discharged on June 16.

Based upon the weekly prevalence, the epidemic curve showed three peaks with the outbreak reaching the high number of colonization cases when the NICU was near to the maximum of its bed capacity (Figure 1).

Detection of the epidemic *E*. *coli* strain was not performed on the staff members or the environment.

The univariate analysis identified only a lower birth weight as a risk factor associated with the acquisition of ESBL-*E. coli* (cases vs. controls, 1,963 vs. 2,522 g, P = 0.02). In contrast, transfer from another hospital, device use, treatment with ampicillin-sulbactam and gentamicin proved not to be risk factors. There were no statistically significant differences in the length of stay between cases and controls (median 14 vs.10, range 1–78 vs. 3–155 days, *P* = 0.41).

At least two rectal swabs were cultured from 13 out of the 15 infants colonized by ESBL-*E.coli*. The ESBL-*E.coli* isolates were susceptible to carbapenems, gentamicin and colistin. They were resistant to amoxicillin-clavulanate, cefotaxime, ceftazidime, ceftriaxone, cefepime, ciprofloxacin and levofloxacin. ESBL-*E. coli* was attributed with phylogenetic group B2.

PCRs for *bla*_CTX-M_ and *aac*(*6*’)*-Ib-cr* genes were positive. Sequencing identified the presence of *bla*_CTX-M-15_. Resistances proved to be located on conjugative plasmids carrying FII and FIA replicons, able to transfer the whole pattern of resistance to the recipient *E. coli* strain. All isolates were analyzed by rep-PCR, showing a >95% similarity. MLST analysis attributed isolates to ST131.

### Infection control interventions

ESBL-*E. coli* colonized infants were placed under contact precautions. Cohort care was implemented by designated healthcare workers (HCWs), but colonized infants were not physically segregated after consideration of facility design and ward staffing issues. The decision to avoid overcrowding was also made on March 27 by restricting the admission of new patients to the ward (Figure 1). Thorough sanitization of surfaces, equipment and caring devices was carried out. All NICU nursing and medical personnel were informed of the outbreak and training sessions on infection control practices and modes of transmission of ESBL-*E. coli* were arranged. Restriction of third generation cephalosporins and use of carbapenems for infants showing suspected late-onset sepsis were implemented. No further patients meeting the case definition were observed after June 2012.

## Discussion

The global rise of MDR organisms is a serious public health threat. The influx in NICUs of community-associated MDR organisms is of special concern and jeopardizes the effectiveness of prevention and control of measures tailored on healthcare-associated organisms. Sources and routes of entry, indeed, may be different with a more significant role of community reservoirs, such as mothers and HCWs. Strategies to prevent and control these organisms should likely differ as well.

We report an outbreak of colonization in a NICU by an ESBL-*E.coli* strain. A similar event has been infrequently reported. Indeed, a cluster of neonatal invasive infections by CTX-M-15 producing *E. coli*, two of which lethal, was detected in 2005 in Algeria [13]. One further fatal case of neonatal meningitis due to a CTX-M-15 producing strain has been described in France in 2005[14]. Two additional outbreaks have been reported in 2010 in Switzerland and Spain, respectively, caused by TEM-52 and CTX-M-14 producing strains [4,5].

The ESBL*-E.coli* detected in our investigation proved to belong to the international hyperepidemic ST131 phylogroup B2 clone [1]. Accordingly, it showed plasmid-carried *bla*_CTX-M-15_ and *aac*(*6*’)*-Ib-cr* resistance determinants. In recent years, plasmid mediated resistances to β-lactam antibiotics and fluoroquinolones have become increasingly prevalent in community-onset *E. coli* infections [1,2]. The epidemiology of ST131 in Italy is poorly characterized and can be inferred only by a few limited studies. Twenty-four percent out of 148 fluoroquinolone-resistant *E.coli* from uncomplicated urinary tract infections in eight European countries were attributed to the ST131 clone, with a proportion of 34.4% within the Italian isolates [2]. Moreover, ST131 clone was found to be prevalent among 64 ciprofloxacin-resistant extra-intestinal isolates in Rome, Italy, in 2006 [15]. Though poorly representative, these data suggest that ST131 is likely circulating in the community in our geographic area.

Within the NICU patients under investigation, no cases of infection due to the outbreak *E. coli* strain occurred in the study period. This proves that clinically silent intestinal colonization may contribute to the persistence and spread of MDR Gram negative pathogens in NICU. Detection of these events may be delayed or missed completely if no active surveillance is in place and culture results from clinical specimens are the primary means of identifying patients carrying these organisms [16]. Moreover, three subjects had intermittent recovery of ESBL-*E.coli*, which can be associated with carriage at concentrations below the detection limit for rectal swab specimens. This has been previously documented in epidemiological studies conducted in healthcare settings, supporting the need for multiple cultures prior to discontinuation of contact precautions or, alternatively, the advisability to label as continuously colonized a patient following the first isolation [17]. Of interest, infants discharged from NICU have been recently demonstrated to be possible long-term fecal carriers of ESBL-producing enterobacteria and to represent a significant reservoir for intra-household spread of ESBLs [18].

In our experience, attempts were no performed to detect an environmental contamination or carriage by HCWs. Indeed in many MDR Gram negative outbreaks, the stools of colonized infants have proved to be the reservoir for the continued dissemination of the outbreak strains [19,20]. In addition, environmental screening is rarely useful for MDR *Enterobacteriaceae*, as the sensitivity is generally low and the risk of a given site, such a surface or a medical equipment, acting as a reservoir is unpredictable [21]. Because the pattern of acquisition suggested a cross-transmission between infants rather than a common point-source, priority was given to measures aimed at interrupting the transmission chain. On the other hand, previous outbreaks have been traced to contaminated breast milk [3], or perinatal exposure during vaginal delivery [5], both events being unlikely in our situation because the index case was born by caesarean section and exclusively formula-fed. However, the medical history of this infant suggests that the outbreak ESBL-*E. coli* strain was more likely first acquired into the NICU and then disseminated via cross-transmission.

According with previous reports, ESBL-*E. coli* colonization was associated with lower birth weight. No further risk factors were identified. It is likely that spread by cross-transmission was occurring regardless of the different prevalence of possible exposures at risk between cases and controls.

## Conclusions

Our report highlights the need to strictly monitor the spills in the NICU setting of MDR community-associates strains. As β-lactam and fluoroquinolone resistant *E. coli* strains are increasingly spreading in the community, this event could become more frequent. In our case, invasive infections did not develop in any of the 15 neonates. However, serious or fatal infections have been previously described [3,4,9,10]. More information about the prevalence of ESBL-*E.coli* in the community is needed to accurately assess and manage this risk in NICU.

## Abbreviations

ESBL: Extended spectrum β-lactamases; E. coli: *Escherichia coli*; ST: Sequence type; NICU: Neonatal intensive care unit; MDR: Multidrug resistant; EUCAST: European committee on antimicrobial susceptibility testing; PCR: Polymerase chain reaction; MLST: Multilocus sequence typing; HCW: Healthcare worker.

## Competing interests

The authors declare that they have no competing interests.

## Authors’ contributions

MG, DC, GC and CM designed and supervised the study and drafted the manuscript. DMG was in charge of isolation, identification and susceptibility testing. AA and CB were in charge of molecular typing. SD, DMG and FN participated to the surveillance program, and contributed to the interpretations of results. All authors have read and approved the final manuscript.

## References

[B1] Rogers BA Sidjabat HE Paterson DL *Escherichia coli* O25b-ST131: a pandemic, multiresistant, community-associated strain J Antimicrob Chemother 2011 66 1 14 10.1093/jac/dkq415 21081548

[B2] Cagnacci S Gualco L Debbia E Schito GC Marchese A European emergence of ciprofloxacin-resistant *Escherichia coli* clonal groups O25:H4-ST131 and O15:K52:H1 causing community-acquired uncomplicated cystitis J Clin Microbiol 2008 46 2605 2612 10.1128/JCM.00640-08 18579721 PMC2519467

[B3] Giuffrè M Cipolla D Bonura C Geraci DM Aleo A Di Noto S Nociforo F Corsello G Mammina C Epidemic spread of ST1-MRSA-IVa in a neonatal intensive care unit, Italy BMC Pediatr 2012 12 64 10.1186/1471-2431-12-64 22682025 PMC3407518

[B4] Moissenet D Salauze B Clermont O Bingen E Arlet G Denamur E Mérens A Mitanchez D Vu-Thien H Meningitis caused by *Escherichia coli* producing TEM-52 extended-spectrum β-lactamase within an extensive outbreak in a neonatal ward: epidemiological investigation and characterization of the strain J Clin Microbiol 2010 48 2459 2463 10.1128/JCM.00529-10 20519482 PMC2897521

[B5] Oteo J Cercenado E Fernández-Romero S Saéz D Padilla B Zamora E Cuevas O Bautista V Campos J Extended-spectrum-β-lactamase-producing *Escherichia coli* as a cause of pediatric infections: report of a neonatal intensive care unit outbreak due to a CTX-M-14-producing strain Antimicrob Agents Chemother 2012 56 54 58 10.1128/AAC.05103-11 21986825 PMC3256038

[B6] Tschudin-Sutter S Frei R Battegay M Hoesli I Widmer AF Extended spectrum ß-lactamase-producing *Escherichia coli* in neonatal care unit Emerg Infect Dis 2010 16 1758 1760 10.3201/eid1611.100366 21029537 PMC3294509

[B7] Mammina C Di Carlo P Cipolla D Giuffrè M Casuccio A Di Gaetano V Plano MR D'Angelo E Titone L Corsello G Surveillance of multidrug-resistant gram-negative bacilli in a neonatal intensive care unit: prominent role of cross transmission Am J Infect Control 2007 35 222 230 10.1016/j.ajic.2006.04.210 17482993

[B8] European Committee on Antimicrobial Susceptibility Testing (EUCAST EUCAST breakpoint table version 1.0 Available at: http://www.eucast.org. (last accessed 17 October 2012)

[B9] Clermont O Bonacorsi S Bingen E Rapid and simple determination of the *Escherichia coli* phylogenetic group Appl Environ Microbiol 2000 66 4555 4558 10.1128/AEM.66.10.4555-4558.2000 11010916 PMC92342

[B10] Dallenne C Da Costa A Decré D Favier C Arlet G Development of a set of multiplex PCR assays for the detection of genes encoding important β-lactamases in *Enterobacteriaceae* J Antimicrob Chemother 2010 65 490 495 10.1093/jac/dkp498 20071363

[B11] Carattoli A Bertini A Villa L Falbo V Hopkins KL Threlfall EJ Identification of plasmids by PCR-based replicon typing J Microbiol Methods 2005 63 219 228 10.1016/j.mimet.2005.03.018 15935499

[B12] Lau SH Cheesborough J Kaufmann ME Woodford N Dodgson AR Dodgson KJ Bolton EJ Fox AJ Upton M Rapid identification of uropathogenic *Escherichia coli* of the O25:H4-ST131 clonal lineage using the DiversiLab repetitive sequence-based PCR system Clin Microbiol Infect 2010 16 232 237 10.1111/j.1469-0691.2009.02733.x 19416293

[B13] Ramdani-Bouguessa N Mendonça N Leitão J Ferreira E Tazir M Caniça M CTX-M-3 and CTX-M-15 extended-spectrum β-lactamases in isolates of *Escherichia coli* from a hospital in Algiers Algeria 2006 44 4584 4586 10.1128/JCM.01445-06 PMC1698418 16988017

[B14] Boyer-Mariotte S Duboc P Bonacorsi S Lemeland JF Bingen E Pinquier D CTX-M-15-producing *Escherichia coli* in fatal neonatal meningitis: failure of empirical chemotherapy J Antimicrob Chemother 2008 62 1472 1474 10.1093/jac/dkn362 18772159

[B15] Cerquetti M Giufrè M García-Fernández A Accogli M Fortini D Luzzi I Carattoli A Ciprofloxacin-resistant, CTX-M-15-producing *Escherichia coli* ST131clone in extraintestinal infections in Italy Clin Microbiol Infect 2010 16 1555 1558 10.1111/j.1469-0691.2010.03162.x 20121822

[B16] Simon A Tenenbaum T Surveillance of multidrug-resistant Gram-negative pathogens in high risk neonates – does it make a difference? Ped Infect Dis J 2013 10.1097/INF.0b013e3182875227 23340567

[B17] Weintrob AC Roediger MP Barber M Summers A Fieberg AM Dunn J Seldon V Leach F Huang XZ Nikolich MP Wortmann GW Natural history of colonization with gram-negative multidrug-resistant organisms among hospitalized patients Infect Control Hosp Epidemiol 2010 31 330 337 10.1086/651304 20175687

[B18] Löhr IH Rettedal S Natås OB Naseer U Oymar K Sundsfjord A Long-term faecal carriage in infants and intra-household transmission of CTX-M-15-producing *Klebsiella pneumoniae* following a nosocomial outbreak J Antimicrob Chemother 2013 10.1093/jac/dks502 23288401

[B19] Diekema DJ Barr J Boyken LD Buschelman BJ Jones RN Pfaller MA Herwaldt LA A cluster of serious *Escherichia coli* infections in a neonatal intensive-care unit Infect Control Hosp Epidemiol 1997 18 774 776 10.1086/647536 9397375

[B20] Almuneef MA Baltimore RS Farrel PA Reagan-Cirincione P Dembry LM Molecular typing demonstrating transmission of gram-negative rods in a neonatal intensive care unit in the absence of a recognized epidemic Clin Infect Dis 2001 32 220 227 10.1086/318477 11170911

[B21] Siegel JD Rhinehart E Jackson M Chiarello L Healthcare Infection Practices Committee Management of multidrug-resistant organisms in health care settings Am J Infect Control 2007 35 S165 S193 10.1016/j.ajic.2007.10.006 18068814

